# Motivation and self-efficacy in cycling and running athletes: a person-centered approach

**DOI:** 10.3389/fpsyg.2025.1533763

**Published:** 2025-05-02

**Authors:** Elżbieta Tokarska, Aleksandra M. Rogowska

**Affiliations:** Institute of Psychology, Faculty of Social Sciences, University of Opole, Opole, Poland

**Keywords:** cluster analysis, cyclists, cycling athletes, runners, self-efficacy, sports motivation

## Abstract

**Introduction:**

The study aims to examine the motivation and self-efficacy of athletes who are practicing cycling and running using the person-centered approach.

**Methods:**

A sample of 156 professional athletes (73 cyclists and 83 runners), including 65% of men, participated in the cross-sectional study. The mean age of athletes was 32 years old, ranging from 18 to 64 (*M* = 31.68, *SD* = 11.26). The online survey included the Sports Motivation Scale (SMS-28) and the Generalized Self-Efficacy Scale (GSES) to assess self-reported motivation for sports activity and the general sense of self-efficacy.

**Results:**

The K-means cluster analysis identified three groups of athletes based on their scores in sports motivation and self-efficacy. The first sample included “Internally motivated athletes,” who scored high in self-efficacy and three scales of intrinsic motivation (to know, to accomplish, and to experience stimulation) and simultaneously scored low in three scales of external motivation (introjected, identified, and external regulation), and amotivation. The second group comprised “Externally motivated athletes,” scoring high in all dimensions of extrinsic motivation while low in intrinsic motivation scales and self-efficacy. The third group of “Highly motivated athletes” scored high on self-efficacy and all dimensions of sports motivation. The multivariate analysis of variance (MANOVA) and one-way ANOVA showed several differences in sports motivation and self-efficacy between particular clusters.

**Discussion:**

Classifying athletes into three groups based on their motivation and self-efficacy can be utilized in sports psychology. In particular, those externally motivated athletes require psychological support to increase their intrinsic motivation and self-efficacy.

## 1 Introduction

Achieving success in sports necessitates that athletes possess a range of skills, including concentration, stress management, and the ability to perform optimally under challenging and demanding environmental conditions. Psychological support is deemed essential, as it aids in identifying an athlete's capabilities and limitations, thereby enabling the minimization or maximization of their impact on sports competition. The enhancement of psychosocial skills is identified as the primary and most critical component for maintaining consistent performance at a high level of competition. This study endeavors to investigate the associations between self-efficacy and sports motivation among cycling and running athletes, employing a person-centered approach.

Both self-efficacy and motivation are critical variables in endurance sports, as these disciplines impose significant mental and physical demands on athletes, necessitating sustained effort over time. Motivation to engage in systematic actions may be a pivotal factor during the preparatory phase for competition, while self-efficacy can be instrumental during the competition itself and in shaping athletes' sports profiles. This study aims to identify specific patterns of psychological resources, such as self-efficacy and sports motivation, within a cohort of cyclists and runners. The interaction between self-efficacy beliefs and the configuration of sports motivation dimensions may be essential for determining both current performance and long-term career trajectories in sports.

According to social cognitive theory (Bandura, 1977), self-efficacy is defined as an individual's belief in their capacity to succeed in specific situations or accomplish tasks. This belief is pivotal in determining how individuals approach goals, tasks, and challenges. The enhancement of self-efficacy is influenced by past achievements, vicarious experiences, verbal persuasion, and alterations in the perception of physiological arousal. A meta-analysis has demonstrated a positive correlation between self-efficacy and sports achievements (Moritz et al., 2000). High self-efficacy is associated with a greater propensity to confront and persist through difficulties, thereby enhancing sports performance. In particular, self-efficacy has been shown to be a significant predictor of performance in endurance sports, such as cycling and running, where maintaining a consistent level of physical activity is crucial (Samendinger et al., 2019; Horcajo et al., 2022). In a study of cyclists, self-efficacy was found to increase over time, explaining a larger portion of the variance in performance as the sessions progressed (Samendinger et al., 2019). Similarly, in ultra-marathon runners, self-efficacy was highly related to mental toughness, although it did not directly correlate with performance outcomes in elite competitions (Brace et al., 2020).

Engagement in sports and physical activity is influenced by a variety of factors, including psychological motivations, body image, social interactions, and health benefits. Psychological need satisfaction and achieving a flow state, as well as enjoyment and mastery, plays a crucial role in sports participation and are important for maintaining long-term engagement in physical activities (Sierra-Díaz et al., 2019; Zhou et al., 2019). Participants in extreme sports are motivated by factors such as vertigo and catharsis, in addition to traditional motives like competition and ego (Zhou et al., 2019). Concerns about body image and the desire for fitness are common reasons for participation, especially among young people and university students (Allender et al., 2006; Diehl et al., 2018). Social factors, including affiliation and contact with others, are significant motivators. Engaging in sports provides opportunities for social interaction and building a support network, which is particularly valued by older adults (Allender et al., 2006; Diehl et al., 2018). Maintaining physical health, feeling good, and refreshing the mind are primary motivators for engaging in leisure-time physical activity and club sports among university students (Motevalli et al., 2024), wellbeing is a significant motivating factor across different age groups, particularly for older adults (De Maio Nascimento et al., 2023).

Research showed that competitive cyclists are primarily motivated by goal achievement, competition, and recognition, whereas non-competitive cyclists are more driven by weight concerns and social affiliation esteem (LaChausse, 2006). Road cyclists often prioritize goal achievement and competition, while mountain bikers find life meaning as a significant motivation (LaChausse, 2006). In mass cycling events, motivations such as interest/enjoyment, competence/challenge, and fitness are prevalent (Malchrowicz-Mośko et al., 2019). Notable differences between male and female cyclists were also found (LaChausse, 2006; Malchrowicz-Mośko et al., 2019). Runners in ultra-endurance events are often motivated by their attachment to the event, involvement in the sport, and satisfaction from previous events (Koronios et al., 2016). Runners exhibit various motivation profiles, such as “autonomy achievers” who have high levels of autonomous motivation and engage more frequently in running activities. These profiles are associated with distinct training patterns and can inform interventions aimed at increasing physical activity through running (Skejø et al., 2024).

Self-determination theory (SDT) posits that behavior is driven by intrinsic motivation (engaging in an activity for its inherent satisfaction and enjoyment), extrinsic motivation (engaging in an activity due to external rewards or pressures), or amotivation (Ryan and Deci, 2017; Standage and Ryan, 2020). The theoretical framework posits a continuum of motivation ranging from amotivation, characterized by a lack of motivation, to intrinsic motivation, encompassing various forms of extrinsic motivation, including external regulation, introjected regulation, and identified regulation. Identified regulation involves the acknowledgment of the value and benefits of an activity, even in the absence of inherent enjoyment. Individuals consciously accept the behavior and demonstrate a relative willingness to engage in the activity. For instance, an athlete may comprehend the significance of fitness or team spirit, recognizing how participation in sports contributes to these objectives. Introjected regulation is influenced by internal forces, such as guilt or fear, rendering it less self-determined. Individuals may engage in behavior out of a sense of obligation or to avoid negative emotions, such as shame. For example, an athlete may experience guilt or fear of disappointing parents, coaches, or teammates if performance is suboptimal. Further along the spectrum, external regulation represents the least self-determined form of motivation, where individuals act primarily to meet external expectations, obtain rewards such as trophies or monetary compensation, or receive recognition. At the extreme end of the continuum lies amotivation, where individuals lack the intention or drive to engage in a behavior. They may act without conscious effort or planning, feeling ineffective and lacking control. An athlete may lose interest and no longer feel motivated to continue with the training regimen (Teixeira et al., 2012; Rodrigues et al., 2023).

In contrast to the aforementioned perspective, Chemolli and Gagné (2014) assert that an alternative viewpoint can be adopted in research. They demonstrate that motivation varies in its nature rather than in the degree of autonomy. Consequently, an individual may engage in an activity by employing multiple motives simultaneously or may alternate between motives based on situational factors or previous experiences. They highlight that any motive can be adopted by an individual at any time, irrespective of the continuum of relative autonomy. The questionnaire designed to assess these various types of motivation within sports contexts was developed in the 1990s (Briere et al., 1995; Pelletier et al., 1995) and has been utilized in numerous studies globally. Individuals frequently report high levels of intrinsic motivation, with enjoyment being the primary factor, or identified regulation, wherein they engage in exercise due to an acknowledgment of its significance. In terms of introjected motivation, gender differences are observed; males are more inclined to cite social pressure or the desire for ego enhancement as motivations for exercising, whereas females are more likely to mention guilt as a motivating factor. Introjected regulation, which is driven by ego enhancement or contingent self-worth, is heavily dependent on external environmental support. If not internalized, the environment may be perceived as controlling rather than supportive of autonomy, leading to cessation of engagement once external, contingent factors are removed (Gillison et al., 2009). The fulfillment of three fundamental needs—autonomy, competence, and relatedness—can also contribute to motivation for PA and sports (Almagro et al., 2020; Vasconcellos et al., 2020). According to SDT, individuals possess innate psychological needs, such as autonomy, competence, and relatedness, which, when fulfilled, promote optimal functioning, growth, and wellbeing. Consequently, individuals are autonomously motivated. Conversely, when these needs are unmet or only partially satisfied, individuals are more likely to regulate their behavior based on controlled reasons (Vasconcellos et al., 2020).

Vallerand and Losier (1999) observed that athletes exhibiting elevated levels of intrinsic, integrated, and identified motivation demonstrated superior outcomes, including enhanced affective experiences, improved sportsmanship orientations, and increased persistence in their sport, in comparison to those with controlled motivation (introjected, external). In the context of professional American football, players with high intrinsic motivation experienced an augmentation in self-identity and success, which subsequently influenced their performance. Furthermore, the significance of relatedness with fellow players and fans was emphasized as a factor that bolstered their internal motivation (Clancy et al., 2017; Harrolle and Klay, 2019) reported that intrinsic motivation among competitive athletes is lower than that of recreational athletes, as the presence of external rewards in competitive settings may undermine intrinsic motivation.

The relationship between self-efficacy and motivation has been the subject of extensive investigation in recent years. Social cognitive motivation models (SCMM) integrate the interplay of motivational and cognitive factors influencing academic or athletic performance (Schunk, 1991, 1995; Linnenbrink and Pintrich, 2002). Within the SCMM framework, motivation is conceptualized as a dynamic and multifaceted construct, encompassing critical elements such as self-efficacy, attributions, intrinsic motivation, and goals (Linnenbrink and Pintrich, 2002). Notably, self-efficacy can enhance motivational engagement in goal attainment, mastery, and improved performance (Linnenbrink and Pintrich, 2003; Dogan, 2015; Beri and Stanikzai, 2018). Research indicates that a strong sense of self-efficacy predicts more favorable subsequent motivational outcomes (Schunk and DiBenedetto, 2020, 2021). Specifically, self-efficacy exhibits a robust positive correlation with intrinsic motivation, as individuals with high self-efficacy are more inclined to engage in tasks for their inherent interest and enjoyment (Gan et al., 2023).

Research showed that self-efficacy is associated with overall academic motivation among students (Husain, 2014; Maraghi et al., 2018; Ariff et al., 2021; Shengyao et al., 2024). In particular, studies suggest that higher self-efficacy is associated with lower amotivation and higher intrinsic motivation (Walker et al., 2006; Brown, 2010; Blecharz et al., 2015; Buch et al., 2015; Kheirkhah et al., 2017; Fominykh and Kornienko, 2020; De La Cruz et al., 2021; Gan et al., 2023; Morelli et al., 2023). The relationship between self-efficacy and extrinsic motivation is less clear-cut. Some studies found no significant correlation between self-efficacy and extrinsic motivation (Walker et al., 2006; Brown, 2010; Blecharz et al., 2015), while other research showed negative (Buch et al., 2015; Morelli et al., 2023) or even positive association (Kheirkhah et al., 2017; Fominykh and Kornienko, 2020; Gan et al., 2023). This suggests that the relationship may vary depending on the specific contextual circumstances or population studied. Therefore, an identification of specific patterns of the relationship between self-efficacy and motivation using a person-centered approach may explain the inconsistency in previous research.

Previous research predominantly employed variable-centered (nomothetic or group-based) methodologies, which operate under the assumption that the mean score of a given variable is representative of entire populations, thereby presuming human homogeneity (Saqr et al., 2024). This variable-centered approach utilizes population data to establish generalizable laws or norms applicable to the population. Conversely, the person-centered approach posits the existence of subpopulations with potentially varying parameters. While individuals within these subgroups may exhibit similar levels of specific variables, they display diversity in the interactions among variables, leading to the formation of distinct subgroups or individual profiles (Saqr et al., 2024).

Some previous research has focused on motivation within SDT theory, using the person-centered approach. Analyses of motivational cluster profiles included an examination of the relationship between motivation and physical self-perception in adolescent athletes (Çaglar and Aşçi, 2010; Sahin and Bastik, 2019), the relationship between motivational clusters and dispositional flow in young athletes (Murcia et al., 2007), motivational profiles and burnout in professional athletes (Gustafsson et al., 2018), motivational clusters and physical activity relationship (Friederichs et al., 2015), motivation and mental toughness in connection with goal orientations in elite tennis players (Gustafsson et al., 2018). Other research concerned motivational profiles and achievement goal orientation, the nature of athletic beliefs, perceived competence, and perceived motivational climate (Wang and Biddle, 2001; Chian and Wang, 2008). The number of motivational profiles found in athletes varied from three clusters (Murcia et al., 2007; Friederichs et al., 2015) through four clusters (Chian and Wang, 2008; Çaglar and Aşçi, 2010; Sahin and Bastik, 2019), up to five clusters (Wang and Biddle, 2001; Gustafsson et al., 2018). The inconsistency between these solutions suggests that more research is necessary to explain motivational profiles in the PA context by using various variables, measurement methods, and diverse groups of athletes across various sports disciplines. To our knowledge, no previous studies have examined the relationship between motivation and self-efficacy in professional athletes using cluster analysis. This study addresses a gap in the literature by integrating the SDT motivation approach with the concept of self-efficacy, understood as a relatively stable trait, within a single investigation utilizing a person-centered approach in a sports setting context. Also, applying person-centered approach classifies athletes in groups based on dominant motivation character for each group and thus apply universal intervention for each group.

In vast majority of research papers, motivation is discussed from extrinsic, intrinsic, and amotivation perspective. This paper highlights the significance of intrinsic motivation in fostering commitment to sports, serving as a catalyst that not only promotes participation but also enhances the enjoyment of engaging in sports activities. Motivation and the degree of adopting it internally may indicate the behavior tendencies concerning physical activity (Duncan et al., 2010) or sport participation (Anthony and Rosario, 2023). Some researchers point out the diversity of motives for physical activity within the SDT theory and its progression along the continuum or its hierarchical model (Schüler et al., 2023). Numerous research adopt the perspective of motivation as a link between aspects such as: burnout and engagement in sport (Cresswell and Eklund, 2005; Graña et al., 2021; Groenewal et al., 2021), perfectionism (Stoeber, 2011), injury (Machado et al., 2025) or personality (Vlašić and Ivanišević, 2022) in order to find universal characteristic of motivational process not only in sport. It is worth noting that SDT theory offers also alternative perspective of motivation, which takes into account the satisfaction of three psychological needs: competence, autonomy, and relatedness. However, it is concluded that the three areas contribute to self-determined kind of motivation (De Francisco et al., 2020).

Consistent with the person-centered approach, we assume the heterogeneity of cyclists and runners, acknowledging that they may have different profiles or configurations of sports motives and self-efficacy beliefs. Understanding individual differences in self-efficacy and sports motivation can help sports psychologists and coaches develop training strategies and interventions focused on target groups in professional sports settings. We employed K-means cluster analysis to evaluate the diversity among athletes. This statistical method focuses on person-centered analysis, allowing for the identification of subgroups within a population by grouping individuals based on shared traits across various variables. K-means cluster analysis can reveal significant subgroups, each displaying a distinct arrangement or pattern of the variables being examined. This approach uses multivariate and quantitative data to categorize objects and events based on their similarities. Our current study investigates the variability in a particular configuration of self-efficacy and sports motivation within professional sports environments, specifically among cycling and running athletes. Taking into account previous studies described above (Wang and Biddle, 2001; Murcia et al., 2007; Chian and Wang, 2008; Çaglar and Aşçi, 2010; Friederichs et al., 2015; Gustafsson et al., 2018; Sahin and Bastik, 2019), we assume that there are three to five clusters that exhibit differences in motivational and self-efficacy patterns among athletes representing cycling and running.

## 2 Materials and methods

### 2.1 Study design and procedure

The cross-sectional online study was performed using Google Forms. The eligibility criteria included adults (18 years or older) training in cycling or jogging with a membership status to either a sports club or an association. The study was anonymous and voluntary, and the IRB approved the research project. The sample size was determined a priori using G^*^Power ver. 3.1.9.7 (Faul et al., 2007). For the Student's *t*-test, a minimum of 102 people was expected (51 in each sample of runners and cyclists), considering *p* < 0.05 (α), power 0.80 (1 – β), and medium effect size (Cohen's *d* = 0.50). The required sample size was 159 for one-way ANOVA if assumed three groups, *p* < 0.05 (α), power 0.80 (1 – β), and medium effect size (*f* = 0.25). A minimum sample size of 153 individuals was determined for the global effect of MANOVA, considering three groups, seven response variables, *p* < 0.05 (α), power 0.80 (1 – β), and medium effect size (*f*^2^ = 0.06). Finally, the statistical power of minimum 0.80 for K-means clustering requires a total of 60 people (20 observations per each of the three subgroups), assuming an assured cluster separation of Δ = 4 or greater and subgroups of approximately equal size to detect high accuracy in classifying the group membership of individual observations (Dalmaijer et al., 2022).

A link to an online survey was sent to the sports clubs. If sports clubs agreed to participate in the study, they disseminated the survey via e-mail (private mailing list) or by posting information about the study and the link to the survey on the sports club's Facebook profile. Some of the surveys were carried out through direct contact with running and cycling athletes during competitions. The study involved cyclists from cycling clubs associated with the Polish Cycling Association (Lower Silesian Cycling Association, Kuyavian-Pomeranian Cycling Association, District Cycling Association in Łódz, Lublin Regional Cycling Association, Małopolska Cycling Association, Lubusz Cycling Association, Masovian-Warsaw Cycling Association, Opole Cycling Association, Podkarpacki District Cycling Association, Podlasie Regional Cycling Association, Pomeranian Cycling Association, Warmian-Masurian Cycling Association, Wielkopolska Cycling Association, Silesian Cycling Association, Swietokrzyskie Cycling Association) and members of the Cyloopole, Cyklofun cycling associations as well as participants of cycling competitions. The runners who took part in the study were members of running clubs belonging to the Polish Running Association, members of the athletics section of the Academic Sports Association at the Opole University of Technology, the “Harcownik” running association, and participants of the 10 km street runs.

The data were collected between 3 July 2023 and 6 April 2024. Information about the study and the informed consent form were included on the first page of the survey; therefore, the study was completed if the individual agreed to participate. Initially, 163 people responded to the invitation, but one runner refused to take part in the study, and six people did not meet the criteria for the sports discipline. The final sample included 156 athletes. There were no missing data in the study because responding to all survey questions was mandatory for the study to be completed. *Post-hoc* G^*^Power analysis showed that a sample of 156 participants (including 73 cyclists and 83 runners) indicated a power of 0.93 for Student's *t*-test, 0.80 for ANOVA, 0.81 for MANOVA, and 0.97 for K-means cluster analysis (Faul et al., 2007; Dalmaijer et al., 2022).

### 2.2 Measures

#### 2.2.1 Self-efficacy

The Generalized Self-Efficacy Scale (GSES) is a 10-item scale capturing the strength of an individual's general beliefs, expressing their confidence in coping with difficult situations and obstacles (Schwarzer, 1992; Juczyński, 2000). The GSES has been widely adapted and used in 25 countries across various languages and cultures, demonstrating generally acceptable psychometric properties and global applicability. This scale is one of the most frequently used tools for measuring self-efficacy across various domains, including sports and physical activity (Aizava et al., 2024). Participants rate their response using a Four-point Likert Scale (from “Not at all true” = 1 to “Exactly true” = 4). A higher total score (ranging from 10 to 40) indicates a greater generalized sense of self-efficacy. The internal consistency of the GSES in this study was Cronbach's α = 0.82.

#### 2.2.2 Sports motivation

The Sports Motivation Scale (SMS-28) was developed by Pelletier et al. (1995) and Walczak and Tomczak (2019) as a 28-item measure of motivation in a sports context based on self-determination theory (Ryan and Deci, 2017). The SMS-28 is one of the most widely used tools in research to assess motivation in sports contexts. It has been translated, adapted, and validated in multiple languages, including Czech, Arabic, Spanish, Serbian, and Polish, confirming its reliability and validity in various settings and different cultural contexts. The scale has also been used in diverse populations, such as college athletes, senior athletes, and high school students, demonstrating its applicability across different age groups and competitive levels (Clancy et al., 2017). The SMS-28 contains seven subscales (4-item each), arranged into three dimensions of motivation: intrinsic motivation (To know, To accomplish, and To experience stimulation), extrinsic motivation (Identified, Introjected, and External regulation), and one Amotivation scale. A seven-point response scale (1 = “Does not apply to me at all,” 7 = “Applies to me exactly”) assessed the degree to which the given reasons for practicing sports were met. In the present study, Cronbach's α was as follows: 0.78, 0.76, 0.79, 0.73, 0.68, 0.72, and 0.67, 0.90, and 0.80 for the scales To know, To accomplish, To experience stimulation, Identified, Introjected, External regulation, Amotivation, Intrinsic motivation, and Extrinsic motivation, respectively.

#### 2.2.3 Demographic characteristics of the sample

The demographic characteristic of the sample was assessed using several questions about age (continuous variable), gender (women, men, other), sports discipline (cycling, running), additional sport discipline (trained formerly, currently, or none), years of sport experience (continuous variable), the highest level of competitions, number of days trained during a typical week (ranging 1–7), number of minutes trained on a typical day (continuous variable), and a frequency of participation in competitions during the last season (1–5 times, 5–10 times, above 10 times, or none).

### 2.3 Participants

The study involved 73 cyclists aged 18–57 (*M* = 27.86, *SD* = 11.17) and 83 runners aged 18–64 (*M* = 35.04, *SD* = 10.28), with the majority of men (76.71% of cyclists and 59.04% of runners). The characteristics of the study participants are presented in Table 1. In both groups, the vast majority of athletes used to practice an additional sports discipline or still practice it. Most cyclists declared that they participated in training 6 days a week, while most runners trained 3 days a week. The frequency of competition starts to vary. In the group of cyclists, the highest frequency of starts was over 10 times a season, while in the group of runners, almost half of the respondents chose the frequency of 1–5 times. The level of competitions in which the cyclists took part was the Polish Cup, Polish Championships, and international competitions, while runners took part in competitions in Poland and abroad. The average sports experience was 9 years for both cyclists (ranging from 1 to 30 years, *M* = 9.27, *SD* = 6.89) and runners (ranging from 1 to 32 years, *M* = 9.12, *SD* = 7.90). Runners declared on average 5 days a week of training frequency (ranging from 2 to 7 days a week, *M* = 4.80, *SD* = 1.47), while cyclists declared 4 days a week (ranging from 1 to 7 days a week, *M* = 4.07, *SD* = 1.69). The sample of cyclists trained on average 119 min per week (ranging from 40 to 300 min weekly, *M* = 118.63, *SD* = 48.34), while 68 min per week was noted in the runners' group (ranging from 30 to 230 min weekly, *M* = 67.17, *SD* = 27.81).

**Table 1:** Demographic characteristics of athletes (*N* = 156).

**Variable**	**Categories**	**Cyclist (*****n*** = **73)**		**Runner (*****n*** = **83)**	
		***n***	**%**	***n***	**%**
Gender	Women	17	23.29	34	40.96
	Men	56	76.71	49	59.04
Additional sport discipline	Formerly	30	41.10	15	16.87
	Currently	19	26.03	45	54.22
	None	24	32.88	23	27.71
Training frequency during a typical week	1 day	0	0.00	3	3.61
	2 days	5	6.85	10	12.05
	3 days	13	17.81	23	27.71
	4 days	10	13.70	21	25.30
	5 days	17	23.29	5	6.02
	6 days	20	27.40	10	12.05
	7 days	8	10.96	11	13.25
Frequency of participation in competitions during the last season	1–5 times	22	30.14	40	48.19
	5–10 times	12	16.44	24	28.92
	Above 10 times	36	49.32	19	22.89
	None	3	4.11	0	0.00

### 2.4 Statistical analysis

Initially, the parametric properties of all variables were examined using mean (*M*), standard deviation (*SD*), Median (*Mdn*.), skewness, and kurtosis for self-efficacy and sports motivation, considered as continuous variables. We considered close to a normal distribution of data in the medium size study sample (50 < *n* < 300) since skewness (a measure of the asymmetry) ranged between −0.85 and 2.66, and kurtosis (a measure of “peakedness” of a distribution) ranged between −1.34 and 1.46. As a sensitivity analysis, the Student's *t*-test was performed to examine the differences between cyclists and runners in self-efficacy and motivation. The effect size was assessed using Cohen's *d* statistic.

Three clusters (*k* = 3) were considered in the K-means clustering using Hartigan and Wong's method. The K-means clustering model consisted of eight continuous variables (*n* = 8), including three scales of intrinsic motivation (to know, to accomplish, and to experience stimulation), three scales of extrinsic motivation (identified, introjected, and external regulation), amotivation, and self-efficacy. All variables were standardized prior to the statistical analysis to ensure a fair comparison across different scales. Then, the principal component analysis (PCA) was performed to visualize the data, with the 2-dimensional plane spanned by three cluster centroids and modeled by ball-shaped clusters. Also, the plot of means across clusters was performed to show differences in variables in the model. Finally, the statistical difference between clusters in each variable was assessed using multivariate analysis of variance (MANOVA) for seven scales of motivation and one-way ANOVA for self-efficacy, age, and sports experience. The partial eta square statistic (η^2^_*p*_) was performed to assess effect size, and the Bonferroni *post-hoc* test was carried out to identify statistically significant differences between particular groups. Also, Pearson's χ^2^ test of independence was performed for the comparison of clusters across categories of gender and sports discipline, with Cramer's V as an effect size. All statistical tests were performed using JAMOVI ver. 2.3.28 for Windows.

## 3 Results

### 3.1 Differences between cyclists and runners in self-efficacy and sport motivation

The independent sample of the Student's *t*-test was performed to examine the differences in self-efficacy and motivation, including intrinsic motivation (to know, to accomplish, and to experience stimulation), extrinsic motivation (identified, introjected, and external regulation), and amotivation (Table 2). No intergroup differences were noted, neither for self-efficacy nor for sports motivation.

**Table 2:** Student's *t*-test to assess differences in sports motivation and self-efficacy between cyclists and runners.

**Variable**	**Cyclist(*****n*** = **73)**		**Runner (*****n*** = **83)**		***t*_(154)_**	***p***	***d***
	***M***	***SD***	***M***	***SD***			
Self-efficacy	32.6	4.28	32.02	4.16	0.86	0.394	0.14
Intrinsic motivation	63.33	13.85	64.82	12.44	−0.71	0.480	−0.11
To know	19.45	5.48	19.78	5.33	−0.38	0.703	−0.06
To accomplish	21.59	5.15	21.66	4.51	−0.1	0.924	−0.02
To experience stimulation	22.29	5.23	23.37	3.97	−1.47	0.143	−0.24
Extrinsic motivation	51.25	12.11	51.36	12.07	−0.06	0.953	−0.01
Identified	17.92	5.38	17.45	5.36	0.55	0.584	0.09
Introjected	20.81	4.97	21.42	4.41	−0.82	0.415	−0.13
External regulation	12.52	5.72	12.49	5.89	0.03	0.977	0.01
Amotivation	7.86	3.73	9.08	5.15	−1.68	0.096	−0.27

### 3.2 Cluster analysis for sports motivation and self-efficacy among cycling and running athletes

**Figure 1:**
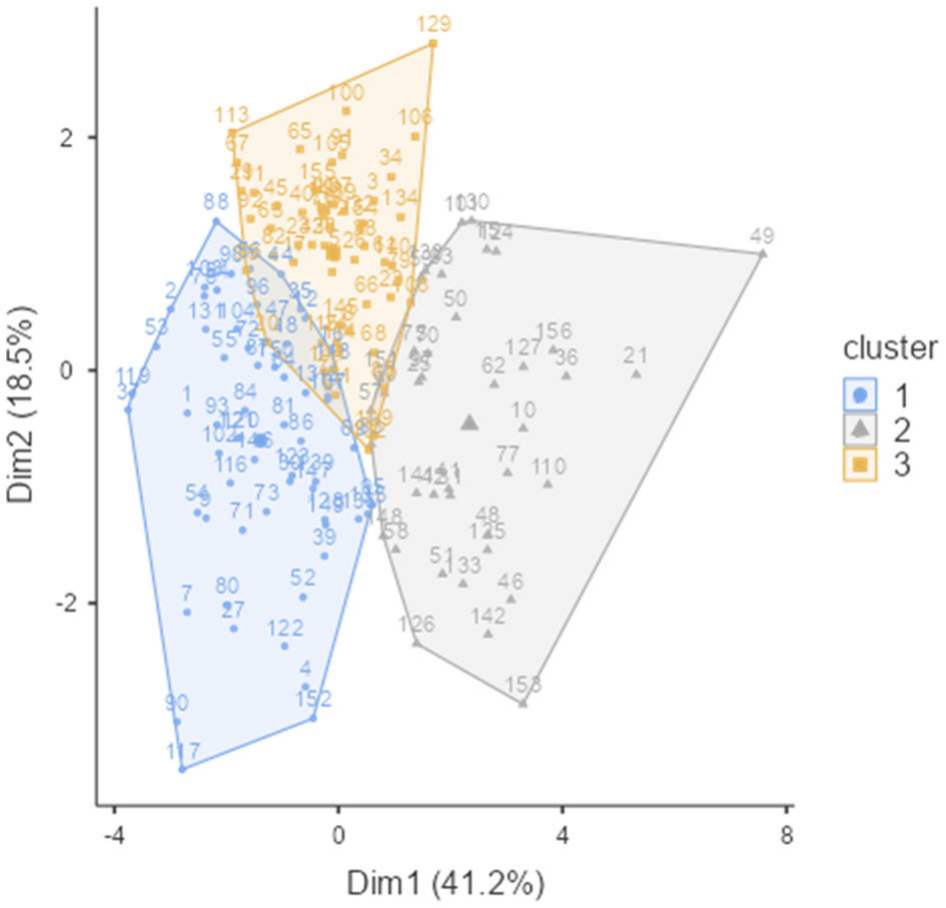
Cluster plot.

The K-means cluster analysis was performed for three clusters, as shown in Table 3 and Figure 1. The prevalence in the first cluster (blue color) included 62 athletes, the second group (in gray) comprised 46 individuals, and the third sample (in orange) consisted of 48 people.

**Table 3:** Centroids of clusters.

**Cluster No**	**Sum of squares**	**IMTK**	**IMTA**	**IMTES**	**EMID**	**EMIT**	**EMER**	**AM**	**SE**
1 (*n* = 62)	284.239	0.360	0.413	0.326	−0.188	−0.225	−0.663	−0.384	0.343
2 (*n* = 46)	291.129	−0.997	−1.176	−1.087	−0.640	−0.406	−0.129	0.159	−0.689
3 (*n* = 48)	218.180	0.491	0.593	0.620	0.856	0.680	0.980	0.344	0.217

IMTK, intrinsic motivation to know; IMTA, intrinsic motivation to accomplish; IMTES, intrinsic motivation to experience stimulation; EMID, extrinsic motivation identified; EMIT, extrinsic motivation introjected; EMER, extrinsic motivation external regulation; AM, amotivation; SE, Self-efficacy.

**Figure 2:**
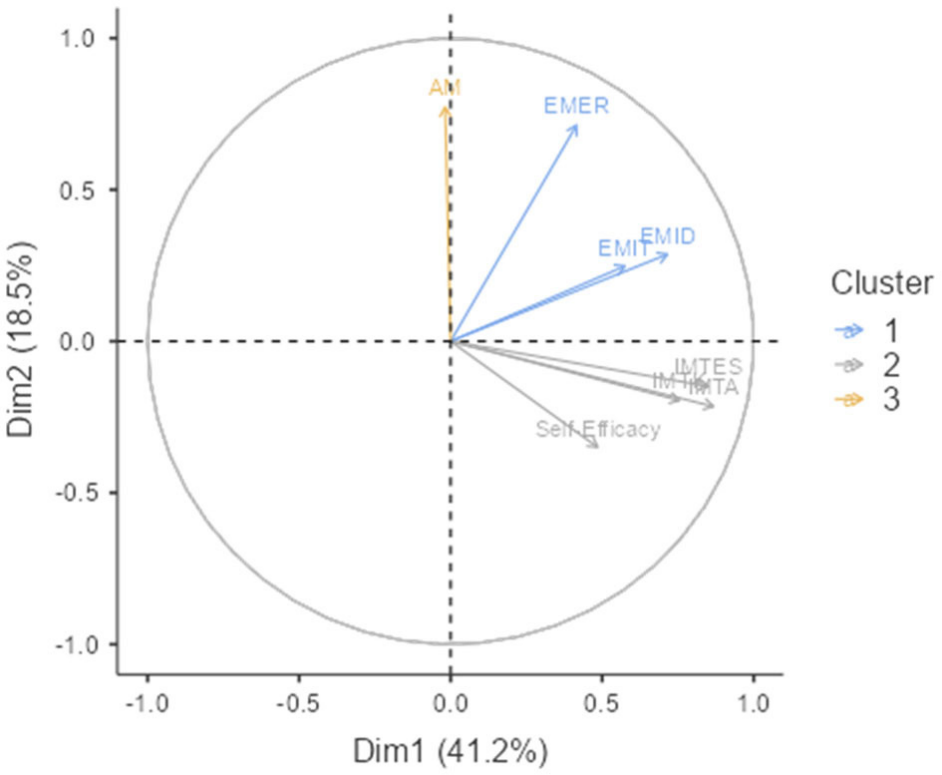
The principal component analysis (PCA) for variables in K-means clustering. IMTK, intrinsic motivation to know; IMTA, intrinsic motivation to accomplish; IMTES, intrinsic motivation to experience stimulation; EMID, extrinsic motivation identified; EMIT, extrinsic motivation introjected; EMER, extrinsic motivation external regulation; AM, amotivation; SE, Self-efficacy.

The PCA analysis (Figure 2) showed that self-efficacy was loaded on the lowest value on axis Y (below 0), together with sequentially all three scales of intrinsic motivation (negative values). Amotivation, in contrast, was presented on the highest value on axis Y (above 0), with all three scales of extrinsic motivation (positive values). Axis Y explains 18.5% of the variance in the clustering model and seems to represent the motivational aspect due to self-determination theory, from believing in a high dependence, sense of certainty, and externally driven behavior (a high value in amotivation) to fully autonomous and internally driven behavior (low value for self-efficacy). The dimension on axis X explains 41.2% of the variance. It may represent the internal need for achievement, with the highest positive value for intrinsic motivation to accomplish and close to 0 (or below) for amotivation. Interpreting these results, we can assume that sports motivation can be explained by the need for achievement (Figure 2).

**Figure 3:**
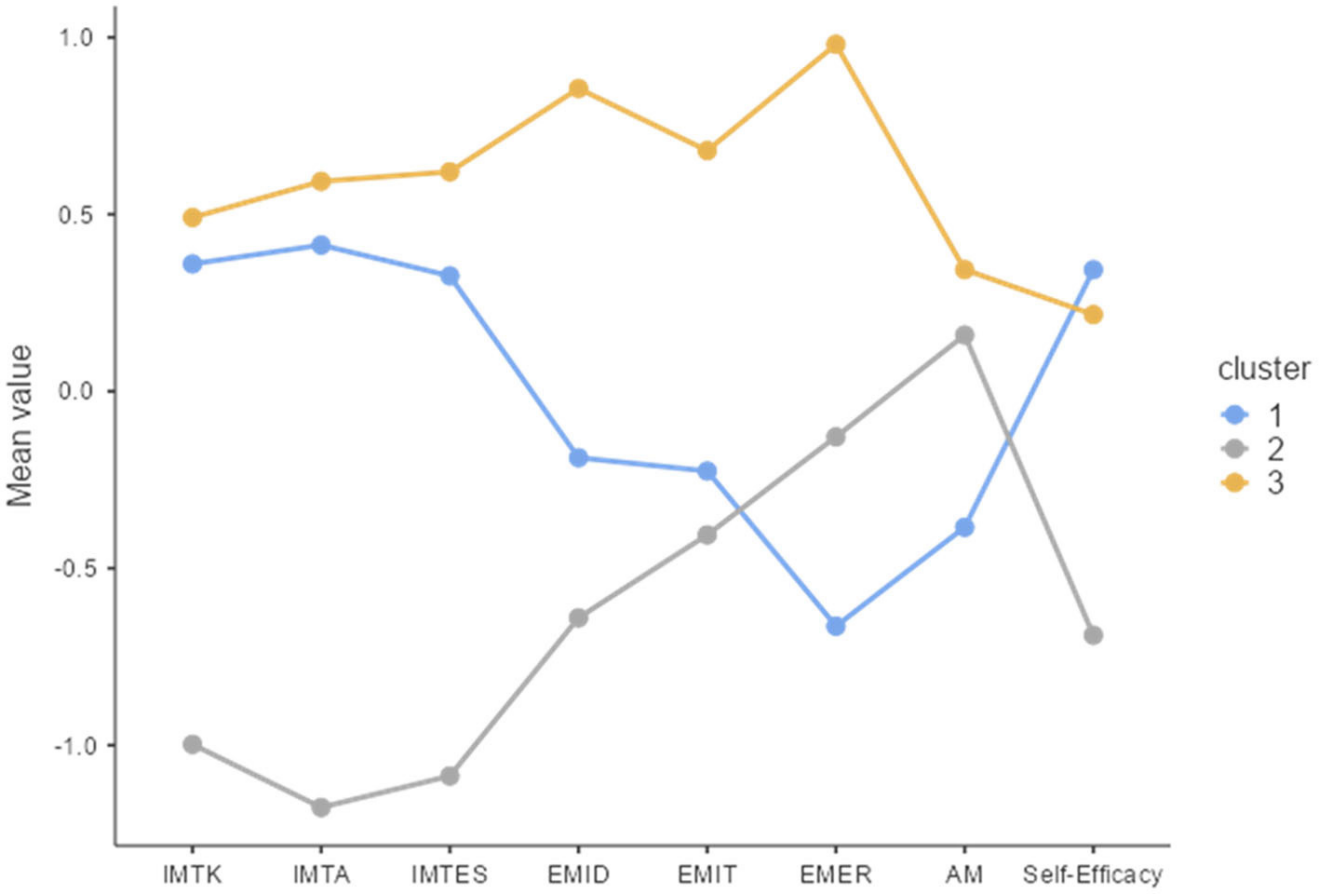
Plot of means across clusters. IMTK, intrinsic motivation to know; IMTA, intrinsic motivation to accomplish; IMTES, intrinsic motivation to experience stimulation; EMID, extrinsic motivation identified; EMIT, extrinsic motivation introjected; EMER, extrinsic motivation external regulation; AM, amotivation.

The mean scores of standardized variables are presented for three clusters in Figure 3. For the first cluster of “Internally motivated athletes” (*n* = 62, blue line), three scales of intrinsic motivation and self-efficacy were relatively high, whereas three scales of extrinsic motivation and amotivation were relatively low. The second cluster of “Externally motivated athletes” (*n* = 46, gray line) showed the opposite pattern to the first cluster, namely relatively high scores in three scales of extrinsic motivation and amotivation, while relatively low scores in three scales of intrinsic motivation and self-efficacy. The third cluster of “Highly motivated athletes” (*n* = 48, orange line), was presented with relatively high scores in all motivational scales and self-efficacy.

### 3.3 Differences between three clusters in sports motivation and self-efficacy

The one-way MANOVA was performed to examine cluster differences in all seven scales of sports motivation, including intrinsic motivation to know, intrinsic motivation to accomplish, intrinsic motivation to experience stimulation, extrinsic motivation identified, extrinsic motivation introjected, extrinsic motivation external regulation, and amotivation. Multivariate analysis showed a significant effect, Wilks' λ = 0.158, *F*_(14, 294)_ = 31.83, *p* < 0.001. There was a significant difference between particular sports motivation scales, *F*_(6, 150)_ = 296.42, *p* < 0.001, η^2^_*p*_ = 0.66, between particular clusters in sports motivation, *F*_(2, 153)_ = 119.52, *p* < 0.001, η^2^_*p*_ = 0.61, and an interaction effect between sports motivation scales and clusters, *F*_(12, 918)_ = 24.877, *p* < 0.001, η^2^_*p*_ = 0.25. The effect size for all of these comparisons was large. A series of one-way ANOVAs were performed to examine differences in sports motivation scales and self-efficacy between particular clusters. Differences between clusters in sports motivation scales and self-efficacy are presented in Table 4.

**Table 4:** The one-way ANOVA for sports motivation scales and self-efficacy.

**Variable**	**Cluster 1 (*****n*** = **62)**		**Cluster 2 (*****n*** = **46)**		**Cluster 3 (*****n*** = **48)**		***F*_(2, 153)_**	**η^2^*_*p*_***	***Post-hoc***
	***M***	***SD***	***M***	***SD***	***M***	***SD***			
IMTK	21.57	3.71	14.26	4.79	22.27	3.93	55.79^***^	0.422	(1 > 2)^***^, (1 = 3), (2 < 3)^***^
IMTA	23.61	2.65	15.98	3.79	24.48	2.93	109.01^***^	0.588	(1 > 2)^***^, (1 = 3), (2 < 3)^***^
IMTES	24.37	2.59	17.85	4.67	25.73	2.18	80.32^***^	0.512	(1 > 2)^***^, (1 = 3), (2 < 3)^***^
EMID	16.66	4.76	14.24	4.51	22.25	3.39	43.48^***^	0.362	(1 > 2)^*^, (1 < 3)^***^, (2 < 3)^***^
EMIT	20.08	4.39	19.24	5.20	24.31	2.43	20.62^***^	0.212	(1 = 2), (1 < 3)^***^, (2 < 3)^***^
EMER	8.66	4.11	11.76	4.62	18.19	3.93	70.19^***^	0.478	(1 < 2)^***^, (1 < 3)^***^, (2 < 3)^***^
AM	6.76	3.09	9.24	4.14	10.08	5.75	8.79^***^	0.103	(1 < 2)^*^, (1 < 3)^***^, (2 = 3)
SE	33.74	4.09	29.39	3.36	33.21	3.78	19.44^***^	0.203	(1 > 2)^***^, (1 = 3), (2 < 3)^***^

IMTK, intrinsic motivation to know; IMTA, intrinsic motivation to accomplish; IMTES, intrinsic motivation to experience stimulation; EMID, extrinsic motivation identified; EMIT, extrinsic motivation introjected; EMER, extrinsic motivation external regulation; AM, amotivation; SE, Self-efficacy. ^*^p < 0.05, ^**^p < 0.01, ^***^p < 0.001.

### 3.4 Differences between three clusters in demographic variables

As a sensitivity analysis, we also compared clusters across age, sports experience, sports discipline (runner, cyclist), and gender (women, men). Differences in age and sports experience between clusters were assessed using one-way ANOVA, whereas cluster differences in sports discipline and age were assessed using Pearson's χ^2^ test of independence. As shown in Table 5, athletes representing three clusters did not differ statistically significantly in age, sports experience, sports discipline, or gender.

**Table 5:** Comparison of clusters for demographic variables.

**Variable**	**Cluster 1 (*****n*** = **62)**		**Cluster 2 (*****n*** = **46)**		**Cluster 3 (*****n*** = **48)**		***F*_(2, 153)_/$\chi_{\left(2\right)}^{2}$**	***p***	**η^2^*_*p*_*/Cramer's V**
	***M/n***	***SD/%***	***M/n***	***SD/%***	***M/n***	***SD/%***			
Age	32.92	10.61	31.13	12.06	30.60	11.37	0.65	0.525	0.01
Sports experience	8.89	7.10	10.30	9.10	8.52	5.90	0.76	0.468	0.01
Sports discipline							1.11	0.574	0.08
Runner	30.00	36.1	25	30.1	28	33.7			
Cyclist	32	43.8	21	28.8	20	27.4			
Gender							4.96	0.084	0.18
Women	26	51	10	19.6	15	29.4			
Men	36	34.3	36	34.3	33	31.4			

## 4 Discussion

The concept of a person-centered approach is not novel. While variable-centered analyses are valuable for discerning individual differences or examining relationships among a limited set of variables within a group, they do not adequately capture the comprehensive patterns of how variables function within individuals. It is crucial to investigate how variables combine and interact within each individual, which constitutes the core of a person-centered approach. The combination of variables can differ among individuals, indicating that some individuals may encounter distinct configurations of variables compared to others. Rather than concentrating exclusively on the variables and their interrelationships within the entire population, person-centered research identifies and compares subgroups of individuals who exhibit similar patterns of variables. Nevertheless, a person-centered approach can complement variable-centered methods and address a unique set of research questions (Meyer et al., 2013).

The study sought to delineate the motivational profiles of athletes through the lens of self-determination theory and self-efficacy measures. This research adopts a novel approach by focusing on athletes engaged in highly demanding endurance disciplines at both regional and national levels. The integration of motivational perspectives with an analysis of their relationship to self-efficacy may serve as a valuable tool for application in professional sports interventions. A non-hierarchical clustering method (k-means), which categorizes observations by employing nearest centroid sorting, was utilized. The study identified the following motivational clusters based on self-determination theory and self-efficacy measures: (1) *internally motivated athletes*, characterized by relatively high scores on three intrinsic motivation scales—namely, motivation to know, to accomplish, and to experience stimulation—as well as self-efficacy; (2) *externally motivated athletes*, who exhibited relatively high scores on three extrinsic motivation scales—identified, introjected, and external motivation—and amotivation, while scoring low on all intrinsic motivation scales and self-efficacy; and (3) *highly motivated athletes*, who demonstrated relatively high scores across all intrinsic and extrinsic motivation scales and self-efficacy, although the mean value of self-efficacy was slightly lower compared to the internally motivated group. Similar to previous studies (Murcia et al., 2007; Friederichs et al., 2015), a three-cluster solution was identified in the data. Contrary to previous studies, it was demonstrated that there exists a group of highly motivated athletes for whom all types of motivation, not solely intrinsic motivation, and self-efficacy were at the highest levels. Furthermore, among a group of externally motivated athletes, amotivation was elevated while self-efficacy was diminished, indicating a potential direction for future intervention.

Furthermore, the comparative analysis of the clusters revealed that Cluster 1 and Cluster 3 exhibited similar levels of intrinsic motivation. However, these two groups diverged in their levels of extrinsic motivation and amotivation, with Cluster 3 displaying higher values in these areas. The self-efficacy values for both clusters were comparable. These findings align with the theoretical framework of the self-efficacy construct, which pertains to individuals' beliefs regarding their capability to accomplish a task. This construct may influence the selection of actions, the exertion of effort, perseverance in goal pursuit, and ultimately, achievement, regardless of whether the goal is driven by internal or external motivations (Bandura, 1977). Previous research (Kheirkhah et al., 2017; Fominykh and Kornienko, 2020; Gan et al., 2023) has demonstrated a positive correlation between self-efficacy and both intrinsic and extrinsic motivation. Furthermore, athletes who possess greater confidence in their abilities tend to exhibit lower levels of demotivation, as evidenced by their low scores on the amotivation subscale. Notably, within cluster 3, introjected motivation achieved the highest value among all extrinsic motives. Teixeira et al. (2012) propose that behavior driven by introjected regulation is rooted in self-approval, leading to anticipated intrapersonal rewards, which may contribute to elevated motivation levels. However, behavior driven by external factors is often short-lived and may result in dropout. This cluster illustrates the multidimensional nature of motivation (Chian and Wang, 2008) and suggests that athletes may display more adaptive behaviors across all motivational scales.

According to SDT, introjected regulation may be perceived as a form of self-regulatory motivation driven by internal pressure and obligation (Vasconcellos et al., 2020). However, it is considered non-autonomous, as the underlying reason for engaging in an activity is externally derived (Chemolli and Gagné, 2014). Concurrently, introjected regulation may impede motivation due to the presence of anxiety and self-criticism. It is positively correlated with need satisfaction, indicating that supportive environments may facilitate its occurrence. Meta-analyses across domains such as exercise, education, public health, work, and sport have corroborated these findings, suggesting potential adaptive and maladaptive outcomes when introjected regulation and other SDT constructs are examined (Vasconcellos et al., 2020).

It is important to consider that intrinsic motivation and identified motivation are often challenging to differentiate, as some adolescents may perceive intrinsic motivation (activities they enjoy) similarly to identified regulation (activities they value). The distinctions between these forms of motivation may be ambiguous, complicating the establishment of their relationship with self-efficacy. Furthermore, introjected regulation and external regulation are collectively categorized as controlled motivation; however, they should be examined as distinct categories, akin to amotivation (Vasconcellos et al., 2020). When intrinsic motivation is evaluated, it is essential to first acquire an internal representation of the outcome, which necessitates prior experience with the goal. The authors highlight that intrinsic motivation can manifest even when outcomes are novel or pertain to uncertain or ambiguous behaviors. Conversely, motivation may be constrained in scenarios involving entirely predictable activities where rewards are anticipated. It is underscored that intrinsic and extrinsic motivation are distinct processes. Their interaction has been demonstrated, raising questions about their dis-sociability. It is noted that both mechanisms are utilized in the pre-decisional deliberation phase of behavioral choice as separate drivers of behavior (Morris et al., 2022).

In light of varying behavioral patterns and outcomes, Chemolli and Gagné (2014) propose that individuals may integrate diverse behavioral regulations, irrespective of the proposed continuum of autonomy. Consequently, the cluster analysis presented aligns with this proposition, as it conceptualizes the motivational profile as a dynamic interplay between motivation and self-efficacy. Person-centered analyses indicate that multi-motivational profiles cultivate different types of motivation, with positive outcomes contingent upon the predominance of autonomous motivation over controlled mechanisms in relation to behavior (Howard et al., 2017).

Despite the limited number of variables, the approach demonstrates significant applicability in sports contexts. Shelly et al. (2020) utilized the k-means clustering technique to categorize elite American football student-athletes, based on physiological data collected over two seasons, into effective training groups with the objective of optimizing the benefits of individualized training approaches. They advocated for the adaptation of training to align with both game demands and athletes' capabilities, which proved to be an efficient aspect of this analysis. This study can also be applied in professional sports by coaches and sports psychologists. The intervention should involve the concurrent enhancement of self-efficacy and intrinsic motivation. Such an intervention is expected to support the achievement of sports success and prevent dropout from physical activity.

While the current study yielded significant findings, certain limitations must be acknowledged to avoid overgeneralization. Primarily, the sample size of cyclists and runners was relatively small, despite comprising only professional athletes. To ensure maximal representativeness among cyclists and runners, invitations were sent to all sports clubs in Poland that associate professional cyclists and runners. However, the response rate was relatively very small. Consequently, the results may not fully represent both disciplines. We also wanted to obtain data from different training stages during one sports season as well as during competitions. Therefore, the data collection lasted 9 months. However, it may be more beneficial to conduct the research at a specific time during the competition season and repeat it during the autumn detraining period to capture the anticipated differences between athletes. A longitudinal study is recommended for future research to thoroughly verify the present results. Further research is required to validate the present findings in a larger and more diverse sample of sports disciplines. Although the SMS-28 scale was well-suited to this research, future studies should employ a revised version of the scale (SMS-6) to broaden the scope of motivation with an integrated perspective. The study was limited to two variables, namely motivation and self-efficacy. Future research should also consider incorporating sports achievements (both lifetime and current) in the profiling of athletes using cluster analysis. Including sports achievements, in addition to motivation and self-efficacy, in the clustering model may provide new insights into the current findings. Since K-means cluster analysis is an unsupervised learning method, the results may not be generalizable to all cyclists and runners. Future studies may use more generative methods of classifying and profiling athletes based on selected variables, such as discriminant analysis or latent profile analysis.

## 5 Conclusions

This study contributes to the existing body of knowledge by demonstrating that the three identified profiles of sports athletes do not merely correspond to three levels of motivation or the basic types of motivation—intrinsic, extrinsic, and amotivation. The research findings indicate that athletes can be categorized into three distinct groups: (1) *highly motivated athletes*, characterized by high levels of self-efficacy and all forms of motivation, ranging from intrinsic motivation to amotivation; (2) *intrinsically motivated athletes*, who exhibit high self-efficacy and intrinsic motivation but low extrinsic motivation; and (3) *lowly motivated athletes*, who display low self-efficacy and intrinsic motivation, coupled with high levels of amotivation and extrinsic motivation. The identified behavioral patterns may serve as valuable criteria for athlete selection and for providing psychological support to those in the third group, characterized by low motivation. The present findings underscore the critical role of enhancing self-efficacy within sports environments to bolster motivation and engagement in physical activities. The outcomes of this research offer insights into evaluating athletes' capabilities in their pursuit of achieving the highest objectives. Given the absence of statistically significant differences concerning age, sports experience, discipline, or gender, it is proposed that interventions could be effectively implemented in a more universal context. As self-efficacy is bolstered by achievements and feedback, such as awards and evaluations from judges and fans, the training process should be structured to afford athletes opportunities for success. This can be achieved by establishing specific long-term objectives, intermediate short-term goals, and a comprehensive action plan. The phased implementation of assumptions, coupled with feedback from a coach, is instrumental in enhancing self-efficacy. At this juncture, cognitive techniques may be introduced, such as imaginative strategies (including relaxation techniques with success visualization), the avoidance of negative thinking and pessimism, and the use of affirmations. Additionally, it is essential to bolster intrinsic motivation by fostering enjoyment and satisfaction derived from physical and sporting activities, setting more effective sporting goals, celebrating achievements, and supporting tasks that lead to development independent of current sports performance and accomplishments.

## Data Availability

The datasets presented in this study can be found in online repositories. The names of the repository/repositories and accession number(s) can be found below: https://data.mendeley.com/drafts/24fw5z3y7c.
